# Human neutrophils drive skin autoinflammation by releasing interleukin (IL)-26

**DOI:** 10.1084/jem.20231464

**Published:** 2024-03-06

**Authors:** Alessia Baldo, Jeremy Di Domizio, Ahmad Yatim, Sophie Vandenberghe-Dürr, Raphael Jenelten, Anissa Fries, Lorenzo Grizzetti, François Kuonen, Carle Paul, Robert L. Modlin, Curdin Conrad, Michel Gilliet

**Affiliations:** 1 Department of Dermatology and Venereology, University Hospital of Lausanne, Lausanne, Switzerland; 2 Division of Dermatology, University of California, Los Angeles, Los Angeles, CA, USA; 3 Department of Dermatology and Venereology, Centre Hospitalier Universitaire, Toulouse, France

## Abstract

Autoinflammation is a sterile inflammatory process resulting from increased neutrophil infiltration and overexpression of IL-1 cytokines. The factors that trigger these events are, however, poorly understood. By investigating pustular forms of psoriasis, we show that human neutrophils constitutively express IL-26 and abundantly release it from granular stores upon activation. In pustular psoriasis, neutrophil-derived IL-26 drives the pathogenic autoinflammation process by inducing the expression of IL-1 cytokines and chemokines that further recruit neutrophils. This occurs via activation of IL-26R in keratinocytes and via the formation of complexes between IL-26 and microbiota DNA, which trigger TLR9 activation of neutrophils. Thus our findings identify neutrophils as an important source of IL-26 and point to IL-26 as the key link between neutrophils and a self-sustaining autoinflammation loop in pustular psoriasis.

## Teaser

Autoinflammation is marked by IL-1 cytokine overexpression and excessive infiltration by neutrophils. By studying pustular psoriasis, we identify a cascade where neutrophils release IL-26 and trigger skin autoinflammation. The mechanism entails IL-26 activation of keratinocytes and concurrently killing of skin microbiota with formation of IL-26–DNA complexes that stimulate TLR9.

## Graphical Abstract

**Figure:**
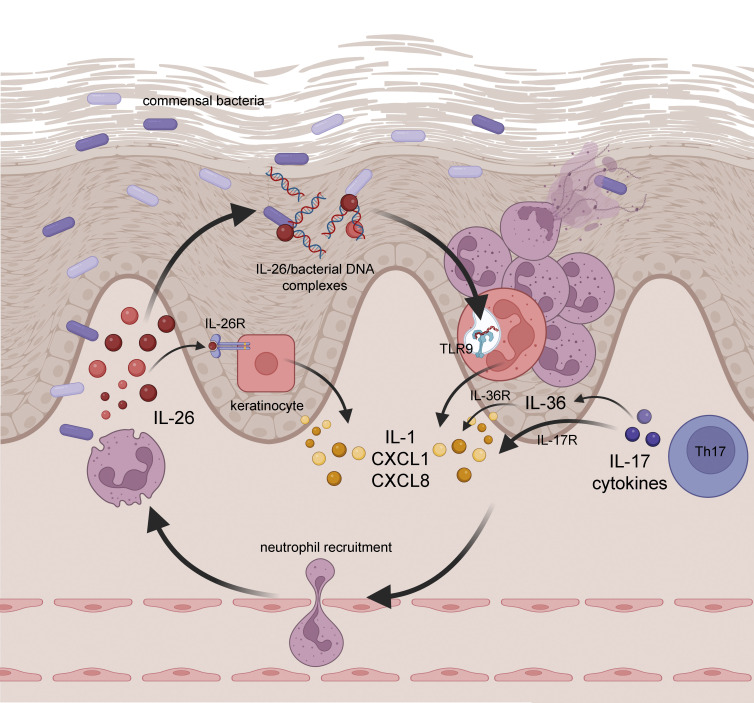

## Introduction

Autoinflammatory disorders are defined by an overactivation of the innate immune system associated with increased production of IL-1 cytokines and “sterile” neutrophil-rich inflammation. While the concept of autoinflammation was originally introduced to describe a group of monogenic diseases characterized by recurrent inflammatory episodes in the absence of infection and without evidence of T or B cell autoimmunity (McDermott et al., 1999; Galeazzi et al., 2006), more recently it has become clear that a number of polygenic inflammatory disorders with prominent neutrophil infiltration are also associated with autoinflammation (Marzano et al., 2018; Satoh et al., 2018). One of these disorders is psoriasis, which classically presents with erythematous scaly plaques driven by Th17 cells (Griffiths et al., 2021; Lowes et al., 2014; Conrad and Gilliet, 2018). Plaque-type psoriasis (PV) displays epidermal hyperproliferation with only modest neutrophil infiltration of skin lesions, mainly in the form of epidermal microabscesses. In contrast, pustular forms of psoriasis show less epidermal thickening, but a predominant neutrophilic skin infiltration with the formation of clinically overt pustules. These pustular forms of psoriasis, which include generalized pustular psoriasis (GPP), palmoplantar pustular psoriasis (PPPP), and acrodermatitis continua of Hallopeau (ACH) (Navarini et al., 2017) are all characterized by high expression levels of IL-1–related cytokines and chemokines that drive neutrophil recruitment to the skin, indicating that they are part of the autoinflammatory spectrum of psoriasis (Johnston et al., 2017; Bachelez, 2018). A role of autoinflammation in the pathogenesis of pustular psoriasis is suggested by the observation that the epidermal accumulation of neutrophils is inhibited by IL-1R blockade in murine psoriasis models (Uribe-Herranz et al., 2013) and by occasional therapeutic responses to IL-1R blockade in GPP, PPPP, and ACH patients (Viguier et al., 2010; Hüffmeier et al., 2014; Tauber et al., 2014; Lutz and Lipsker, 2012; Bachelez et al., 2019). Furthermore, keratinocytes from GPP patients harboring pathogenic loss-of-function mutations in the IL-36 receptor antagonist gene (Marrakchi et al., 2011; Onoufriadis et al., 2011) or gain-of-function mutations in the CARD14 gene (Berki et al., 2015) produce high levels of IL-1 cytokines and neutrophil-recruiting chemokines. However, the factors that trigger and maintain pathogenic autoinflammation in pustular psoriasis remain unclear.

IL-26, an antimicrobial cytokine belonging to the IL-10 family, is expressed in psoriasis, yet the mechanisms by which it contributes to the pathogenesis of the disease are not well understood (Wilson et al., 2007; Meller et al., 2015; Itoh et al., 2019; Hatano et al., 2019). IL-26 exerts potent antimicrobial activity against a broad range of bacteria given its amphipathic and cationic structure, which allows interaction with negatively charged bacterial membranes to increase membrane permeability, leading to bacterial lysis (Meller et al., 2015; Dang et al., 2019; Hawerkamp et al., 2020). IL-26 also exerts proinflammatory functions via the conventional IL-26 receptor expressed by epithelial cells (Hör et al., 2004; Sheikh et al., 2004) or via the formation of complexes with extracellular DNA that activate endosomal TLR9 (Meller et al., 2015), cytosolic cyclic GMP-AMP synthase (cGAS)–stimulator of interferon genes (STING) (Poli et al., 2017; Dang et al., 2019), or the IL-1β inducing inflammasome (Poli et al., 2017) in immune cells. IL-26 is primarily produced by Th17 cells (Meller et al., 2015), but other cellular sources such as synovial fibroblast (Corvaisier et al., 2012), natural killer cells, and macrophages (Fujii et al., 2017), or type 3 innate lymphoid cells (Cols et al., 2016) have been described. The precise mechanism by which IL-26 participates in the pathogenesis of psoriasis is unknown. IL-26–transgenic mice rapidly develop a psoriasis-like skin phenotype upon topical imiquimod treatment via the induction of chemokines known to drive neutrophil recruitment to the skin (Itoh et al., 2019), suggesting a possible link between IL-26 and autoinflammation in psoriasis.

Here, we demonstrate that IL-26 is highly expressed in pustular forms of psoriasis when compared with PV and show that it represents a key driver of autoinflammation and disease activity. IL-26 originates mainly from neutrophils and not T cells, and the high levels of neutrophil-derived IL-26 in pustular psoriasis induce expression of IL-1 cytokines and neutrophil-recruiting chemokines by activating both keratinocytes and neutrophils.

## Results

### IL-26 is highly expressed in pustular psoriasis and associated with neutrophils but not T cells

**Figure 1.**
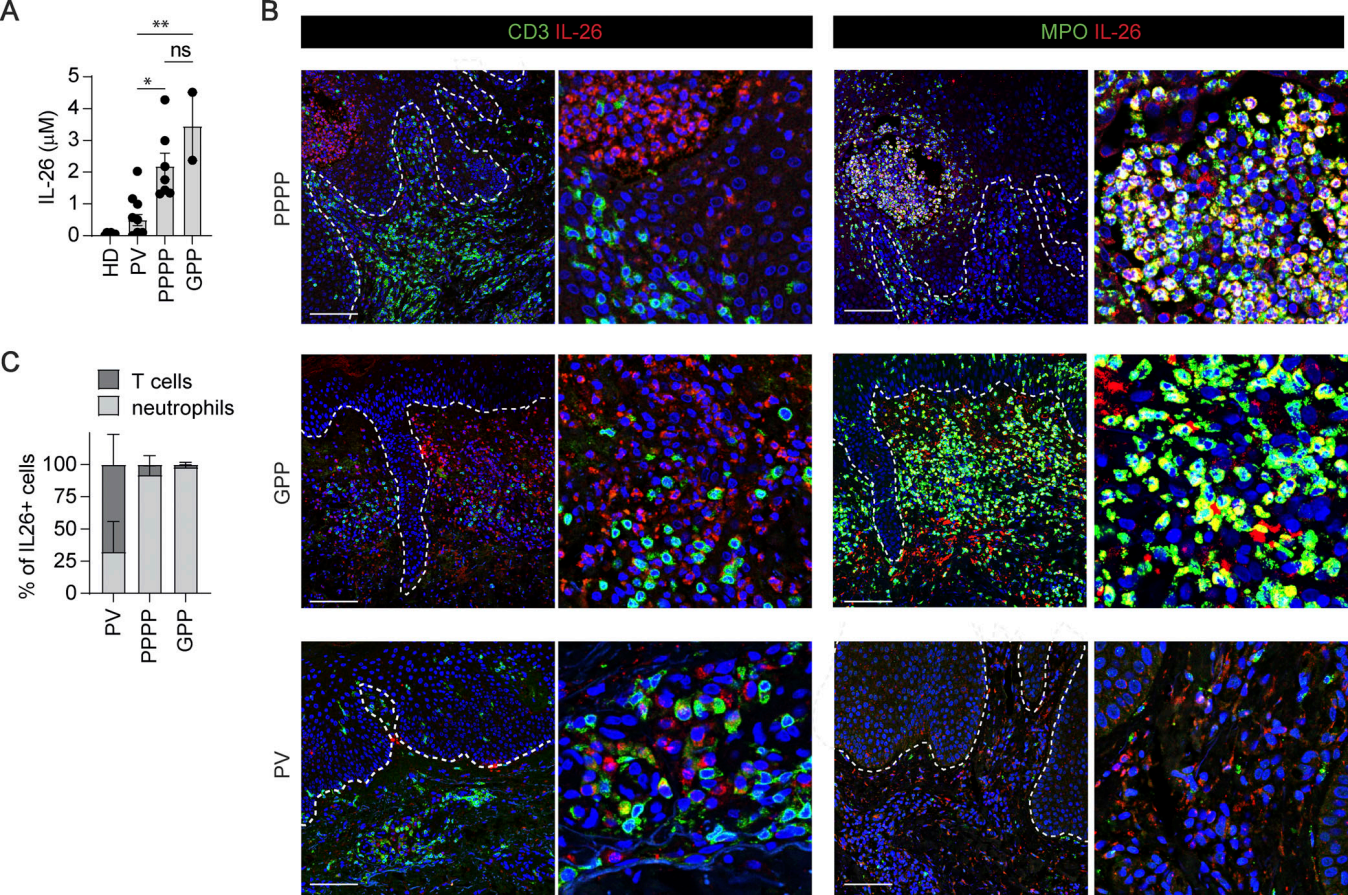
**IL-26 expression in pustular psoriasis is associated with neutrophils and not T cells. (A)** IL-26 protein expression levels in skin lesions of pustular (PPPP, *n* = 7 and GPP, *n* = 2) and PV (*n* = 8) patients, or skin of healthy donors (HD, *n* = 6) as control. Each dot refers to an independent skin donor. **(B)** Confocal microscopy images of skin sections stained with antibodies against IL-26 (red) plus either antibodies against CD3 or against MPO (green), and counterstained with DAPI (blue). Images are representative of three patients. Scale bars are 100 μm. Dashed lines represent the basal membrane. **(C)** Frequency of IL-26–expressing CD3^+^ T cells and MPO^+^ neutrophils in skin sections of PPPP (*n* = 5), GPP (*n* = 3), and PV (*n* = 5). A and B, data represent the mean ± SEM. P values (asterisks) were determined by one-way ANOVA followed by Tukey’s test. *P < 0.05, **P < 0.01.

To investigate whether IL-26 is linked to autoinflammation, we compared IL-26 expression in PV (related to autoimmune T cells) with pustular psoriasis (related to autoinflammation). IL-26 protein was detected in both plaque and pustular forms of psoriasis, but its expression was significantly higher in pustular forms (Fig. 1 A). Tissue immunofluorescence confirmed the increased IL-26 expression in pustular psoriasis (PPPP and GPP) and found a predominant association of IL-26 with neutrophils but not T cells (Fig. 1, B and C). By contrast, IL-26 was predominantly associated with dermal T cells in lesions of PV, which contained only very few neutrophils (Fig. 1, B and C). These data highlight IL-26 overexpression in pustular forms of psoriasis, suggesting a potential link with the autoinflammation process. The findings also suggest that neutrophils, which abundantly infiltrate the skin of pustular psoriasis, represent the principal source of IL-26.

### Neutrophils constitutively express IL-26 and release large quantities more rapidly than T cells

**Figure 2.**
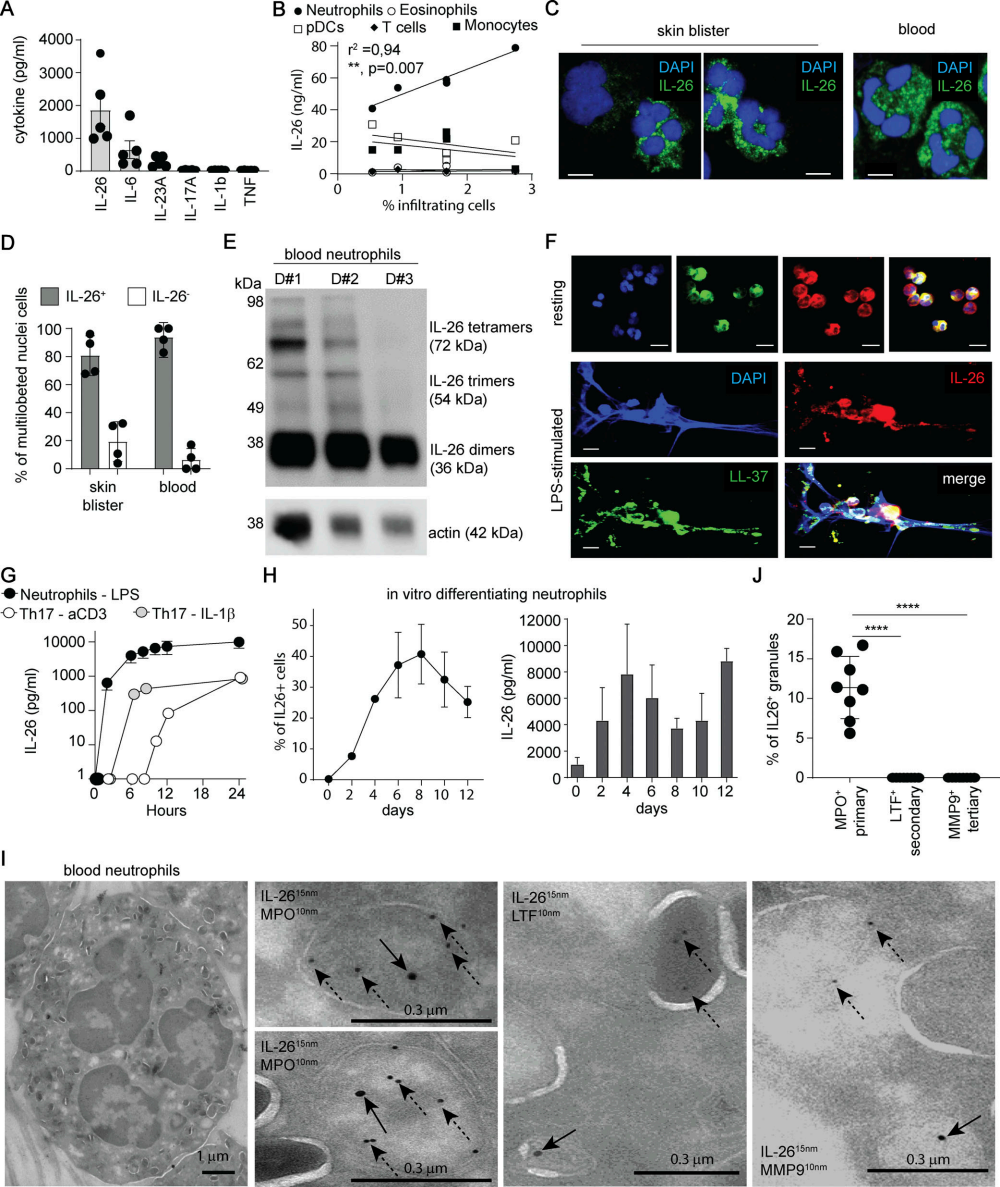
**Neutrophils constitutively express IL-26 and store it in primary granules. (A)** Levels of IL-26, IL-6, IL-23A, IL-17A, IL-1β, and TNF in blister fluids of five healthy donors. Data are shown as mean ± SEM. **(B)** Correlation between the levels of IL-26 and the frequency of neutrophils, eosinophils, monocytes, pDCs, and T cells in the blister fluids. Pearson correlation coefficient and two-tailed statistical significance are indicated. **(C)** Confocal images of cytospinned cells from blister fluids (left and middle) and peripheral blood (right) stained with antibodies against IL-26 (green) and DAPI (blue). Images are representative of five donors. Scale bars are 5 μm. **(D)** Frequency of IL-26–expressing neutrophils identified as cells with multilobulated nuclei in blister fluids and peripheral blood (*n* = 4). Data are shown as mean ± SD. **(E)** IL-26 protein detected by western blot analysis of neutrophil lysates from three independent blood donors. Actin expression served as control. **(F)** Confocal images of blood neutrophils stimulated with LPS, cytospinned, and stained with antibodies against LL-37 (green) or against IL-26 (red), plus DAPI staining (blue). Images are representative of results obtained from three independent donors. Scale bars are 10 μm. **(G)** Kinetics of IL-26 release by blood neutrophils stimulated with LPS or by Th17 cells activated by anti-CD3 beads or by IL-1β. Data are shown as mean ± SD of three donors. **(H)** Frequency of IL-26–expressing cells among differentiating neutrophils measured by immunofluorescence (left) and IL-26 protein levels measured by ELISA of total cell lysates (right). **(I)** TEM images of blood neutrophils stained with gold-particle-labeled antibodies against IL-26 (15 nm) and against MPO, LTF, or MMP9 (10 nm). Solid arrows indicate IL-26 labeling and dashed arrows indicate MPO, LTF, and MMP9 labeling. Images are representative of two donors. Scale bars are shown and represent 1 μm (left image) and 0.3 μm (right images). **(J)** Frequency of IL-26–expressing granules among MPO-, LTF-, or MMP9-expressing granules are shown. Data are the mean ± SD of eight different cells. P values (asterisks) were obtained with one-way ANOVA followed by Tukey’s test. ****P < 0.0001. Source data are available for this figure: SourceData F2.

**Figure S1.**
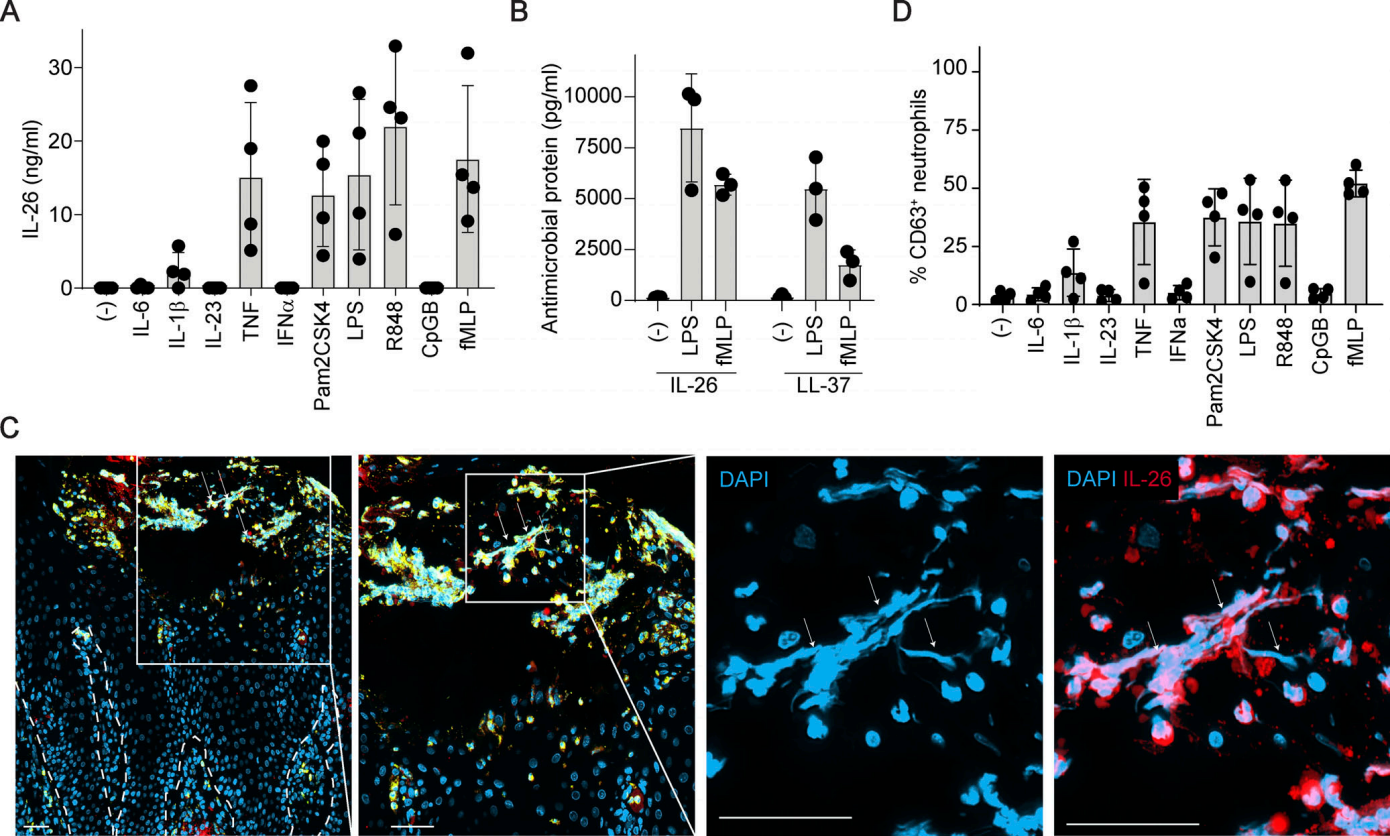
**IL-26 is abundantly released from neutrophil granules and present in NETs. (A)** IL-26 protein released by blood neutrophils following stimulation with indicated cytokines or microbial ligands. Data from four independent blood donors are shown. **(B)** Levels of IL-26 and LL-37 protein released by neutrophils following stimulation with LPS or fMLP. Data obtained from three independent blood donors are shown. **(C)** Confocal microscopy images of pustules representative of five different PPPP skin lesions stained with antibodies against MPO (green) and IL-26 (red), and counterstained with DAPI (blue). NETs are indicated by arrows. Individual stainings and merged images are shown. Zoom-in corresponds to the white box. Scale bars, 50 μm. Dashed line represents the dermo-epidermal junction. **(D)** Flow-cytometry detection of surface CD63 expression in blood neutrophils stimulated overnight with the indicated cytokines or microbial ligands. Data from four independent blood donors are shown.

To confirm that activated neutrophils can produce IL-26, we took advantage of a suction blister model that induces rapid neutrophil recruitment to the skin (Di Domizio et al., 2020). Suction blisters contained high levels of IL-26 but only low levels of other proinflammatory cytokines (IL-6, IL-1β, TNF, and IL-23, and no IL-17A) (Fig. 2 A) and significantly correlated with the presence of neutrophils but not of T cells nor other cells (Fig. 2 B). Immunofluorescence analysis revealed that > 95% of neutrophils, but not other cells, expressed IL-26 (Fig. 2, C and D), indicating that skin-infiltrating activated neutrophils represent the source of IL-26. To determine whether IL-26 expression in neutrophils is dependent on skin infiltration, or whether its expression is already present in resting circulating neutrophils, we analyzed purified blood neutrophils. Like activated blister neutrophils, >95% of resting blood neutrophils already expressed IL-26 (Fig. 2, C and D), suggesting constitutive IL-26 expression by neutrophils. Western blot analysis of lysates confirmed constitutive IL-26 expression by resting blood neutrophils, in the form of dimers, trimers, and tetramers (Fig. 2 E). Stimulation of blood neutrophils with microbial ligands for TLR2, TLR4, TLR8, and the N-formylmethionine-leucyl-phenylalanine (fMLP) receptor induced abundant IL-26 release into the culture supernatants (Fig. S1 A). Importantly, the amount of released IL-26 was comparable with the amount of released LL-37, another major component of the antimicrobial defense system of neutrophils (Fig. S1 B). Furthermore, IL-26 was detected in association with neutrophil extracellular traps (NETs) either generated in vitro or released in vivo in pustular psoriasis (Fig. 2 F and Fig. S1 C).

Because Th17 are the main producers of IL-26, we compared the quantities and the kinetics of IL-26 released by neutrophils with IL-26 produced by equal numbers of Th17 cells. After overnight culture, activated neutrophils released 10-fold more IL-26 than TCR or IL-1β stimulated Th17 cells (Fig. 2 G). Neutrophils released IL-26 more rapidly, with detection as early as 2 h after stimulation, whereas Th17 cells released IL-26 at a slower pace, upon both TCR or IL-1β stimulation (Fig. 2 G). These observations indicate that activated neutrophils release large amounts of IL-26 more rapidly than Th17 cells, which is consistent with the constitutive expression by neutrophils.

### Neutrophils synthesize IL-26 at early differentiation stages and store it as preformed protein in primary granules

**Figure S2.**
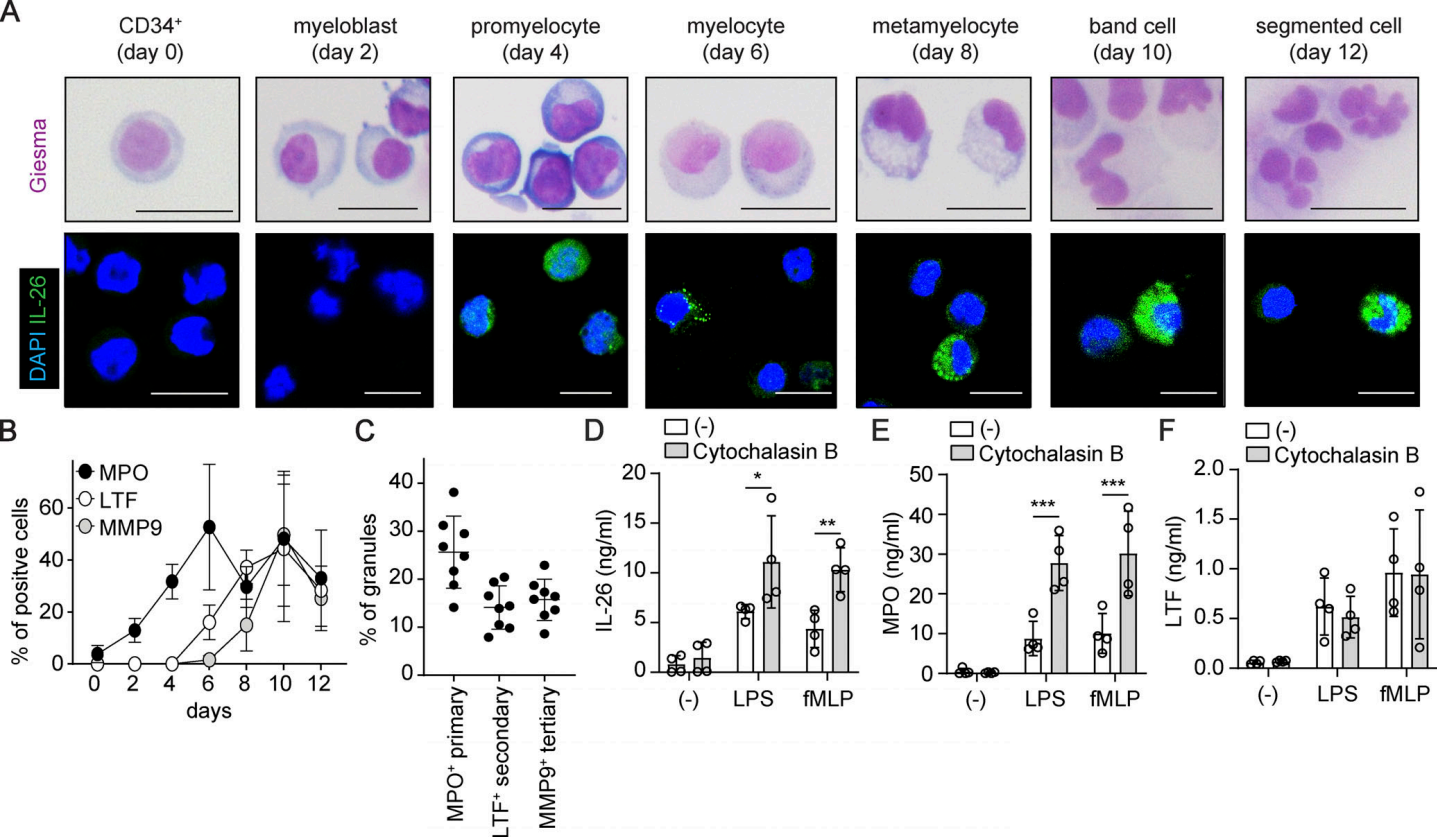
**Neutrophils synthesize IL-26 at early differentiation stages and store it as preformed protein in primary granules. (A)** Images of differentiating neutrophils stained with Giesma (top) and antibodies against IL-26 (green, bottom) during the 12-day neutrophil differentiation process from hematopoietic stem cells. Images are representative of two donors. Scale bars are shown and represent 10 μm. **(B)** Expression of MPO, LTF, and MMP9 in differentiating neutrophils measured by flow cytometry. Data are representative of two independent experiments. **(C)** Frequency of MPO-, LTF-, and MMP9-expressing granules among total cellular granules assessed by electron microscopy. **(D–F)** IL-26 (D), MPO (E), and LTF (F) release by blood-isolated neutrophils left untreated (white boxes) or treated with the actin depolymerizing agent cytochalasin B (gray boxes) and stimulated overnight with LPS or fMLP. P values (asterisks) were obtained with two-way ANOVA followed by Sidak’s test. *P < 0.05, **P < 0.01, ***P < 0.001.

Having found that neutrophils constitutively express IL-26, we next sought to investigate the kinetics of IL-26 synthesis during granulopoiesis. Purified blood hematopoietic stem cells were differentiated into neutrophils in a 12-day culture with SCF, IL-3, and G-CSF, and IL-26 protein expression was quantified by immunofluorescence of intact cells or by ELISA of the cell lysates. In parallel, neutrophil differentiation was monitored based on cell morphology using standard Giemsa staining (Bjerregaard et al., 2003). IL-26 protein was detected in myeloblasts as early as day 2 of culture, reaching significant expression levels in promyelocytes on day 4 (Fig. 2 H and Fig. S2 A). Thereafter, IL-26 expression levels reached a plateau maintained throughout the entire 12-day differentiation process into mature neutrophils (Fig. 2 H and Fig. S2 A). The kinetics of IL-26 expression resembled the kinetics of myeloperoxidase (MPO), a primary granule protein induced at myeloblast/promyelocytic stage, but not the kinetics of lactoferrin (LTF), a secondary granule protein induced at the myelocytic stage of differentiation, nor the kinetics of matrix metallopeptidase 9 (MMP9), a tertiary granule protein, induced later at the band cell stage (Fig. S2 B). As granule proteins are incorporated into their respective granules according to the time of their expression during granulopoiesis, our data suggest that IL-26 is a primary granule protein. To confirm this, we performed transmission electron microscopy (TEM) analysis of the subcellular localization of IL-26 along with MPO, LTF, and MMP9. IL-26 was stained with larger 15-nm-gold-labeled secondary antibodies, whereas MPO, LTF, or MMP9 staining was done with smaller 10-nm-gold-labeled secondary antibodies. Among neutrophil granules, 25.6% ± 7.5 SD were MPO^+^ primary granules, 14.1% ± 4.5 SD were LTF^+^ secondary, and 15.7% ± 4.3 SD were MMP9^+^ tertiary granules (Fig. 2 I and Fig. S2 C). IL-26 was only found in MPO^+^ primary granules but was completely absent in LTF^+^ secondary and MMP9^+^ tertiary granules (Fig. 2, I and J). Hence, neutrophils produce and store IL-26 in primary MPO^+^ granules. In fact, microbial stimuli shown to trigger the release of IL-26 also upregulated surface expression of CD63, a specific marker for degranulation of primary granules (Kuijpers et al., 1991) (Fig. S1 D). Moreover, the actin polymerization inhibitor cytochalasin B, known to enhance primary granule exocytosis, increased the release of IL-26 along with the primary granule protein MPO but did not promote the release of the secondary granule protein LTF from activated neutrophils (Fig. 2, D–F).

### Neutrophil-derived IL-26 triggers skin autoinflammation in pustular psoriasis

**Figure 3.**
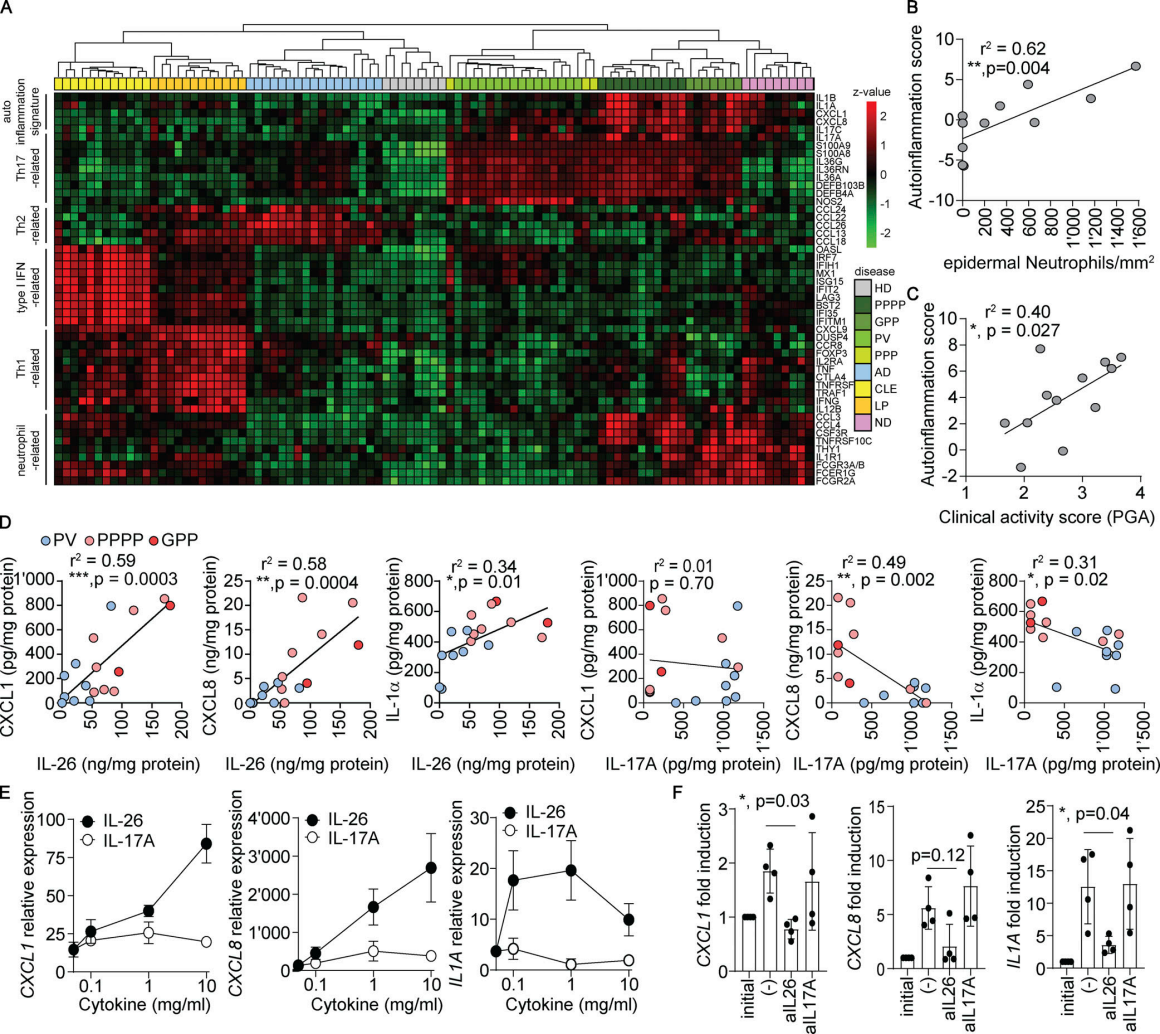
**IL-26 but not IL-17A drives autoinflammation in pustular psoriasis. (A)** Heatmap showing expression of selected immune genes and unbiased clustering of healthy skin (HD, *n* = 8) or lesional skin of PV (*n* = 16), non-pustular palmoplantar psoriasis (PPP, *n* = 3), PPPP (*n* = 11), GPP (*n* = 7), atopic dermatitis (AD, *n* = 17), lichen planus (LP, *n* = 12), discoid lupus erythematosus (CLE, *n* = 12), and neutrophilic dermatoses (ND) including PG (*n* = 3), HS (*n* = 3), and DC (*n* = 3). Color intensity represents the level of normalized gene expression. Immune genes were selected based on differential expression between diseases and categorized into the following inflammation pathways based on known gene function: autoinflammation, Th17 related, Th2 related, type I IFN related, Th1 related, and neutrophil related. **(B)** Correlation between the autoinflammation signature score, calculated as mean expression of *CXCL1*, *CXCL8*, *IL1A*, *IL1B*, and the number of epidermal neutrophils in psoriatic skin samples (*n* = 11). **(C)** Correlation between the autoinflammation signature score and the pustular psoriasis disease activity, measured using the PGA score. **(D)** Correlation between the levels of IL-26 or IL-17A protein and the levels of CXCL1, CXCL8, and IL-1α protein in the lysates of PV (*n* = 8, blue), PPPP (*n* = 7, pink), and GPP (*n* = 2, red) skin lesions. Pearson correlation coefficient and a two-tailed statistical significance are given. **(E)** *CXCL1*, *CXCL8*, and *IL1A* mRNA expression in healthy skin explants stimulated overnight with increasing concentrations of IL-26 or IL-17A. Data are from one out of two experiments with independent donors, and are shown as mean ± SD of triplicate wells. **(F)** *CXCL1*, *CXCL8*, and *IL1A* mRNA expression in pustular psoriatic skin cultured overnight in the presence of blocking antibodies against IL-26 or IL-17A. Data represent fold induction over the expression levels measured in the initial snap-frozen skin biopsy. P values were calculated by one-way ANOVA followed by Tukey’s test.

**Figure S3.**
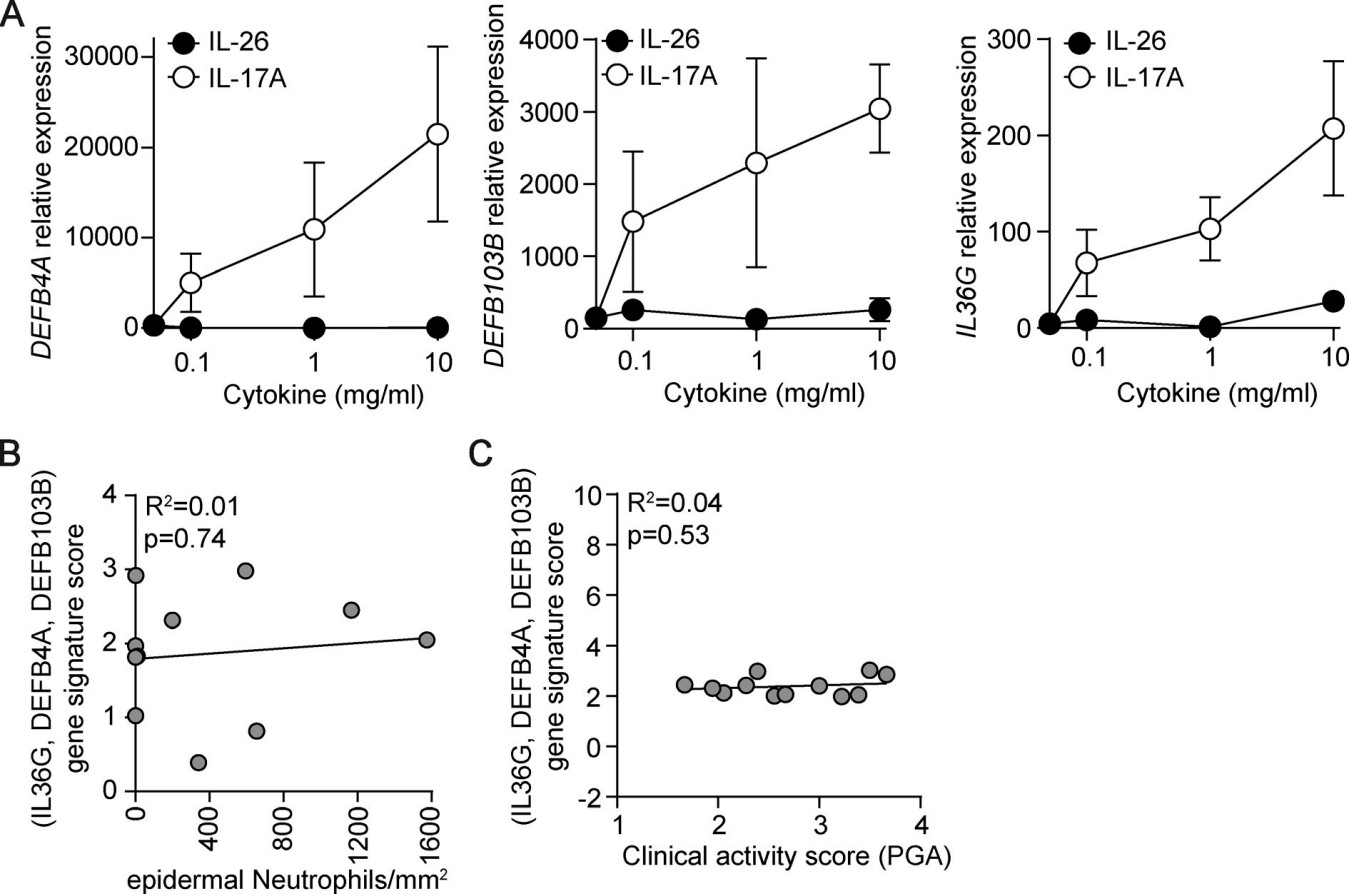
**The IL-17A–induced gene signature is not linked to neutrophil infiltration nor clinical activity in pustular psoriasis. (A)** IL36G, DEFB4A, and DEFB103B mRNA expression in healthy skin explants stimulated overnight with increasing concentrations of IL-26 or IL-17A. The mean ± SD of triplicates is shown and represents the results obtained from two independent donors. **(B)** Correlation between the number of epidermal neutrophils and the IL-17A–related gene signature (IL36G, DEFB4A, DEFB103B) in psoriasis skin (*n* = 11). **(C)** Correlation between the clinical activity score in PPPP patients (assessed by PGA) and the IL-17A–related gene signature in psoriatic skin (*n* = 12).

Having found that IL-26 released by neutrophils is overexpressed in pustular psoriasis, we sought to investigate whether it is linked to the autoinflammation process in pustular psoriasis. Comparison of gene expression profiles from multiple inflammatory skin diseases revealed that pustular psoriasis samples clustered in the vicinity of PV based on a common Th17 signature (*IL17A/C*, *S100A8/A9*, *IL36G/A/RN*, *DEFB4A*, *DEFB103B*), but also identified an additional autoinflammation signature with expression of IL-1 family cytokines *IL1A* and *IL1B* and neutrophil-recruiting chemokines *CXCL1* and *CXCL8* (Fig. 3 A). This autoinflammation signature was specific for pustular forms of psoriasis, including PPPP and GPP skin lesions, but was not detected in PV (Fig. 3 A). The autoinflammation signature significantly correlated with both the numbers of neutrophils in the skin (Fig. 3 B) and the disease activity was quantified by a combined clinical score evaluating erythema, pustulation, and desquamation (Fig. 3 C). The signature also correlated with the high levels of IL-26 protein, but not with the expression levels of IL-17A in the lesions (Fig. 3 D), indicating that IL-26 represents a key driver of autoinflammation in psoriasis and pustular disease activity. Exposure of healthy human skin explants to recombinant IL-26 induced autoinflammation with expression of *CXCL1*, *CXCL8*, and *IL1A* (Fig. 3 E). On the other hand, IL-17A was unable to induce such a signature in the skin cultures, but, instead, induced the expression of *DEFB4A*, *DEFB103B*, and *IL36G*, which are hallmark genes of the Th17 signature (Fig. S3 A). This IL-17A–induced Th17 signature was not related to the neutrophil accumulation nor the disease activity in pustular psoriasis (Fig. S3, B and C). Furthermore, skin explants of pustular psoriasis cultured with a neutralizing anti-IL-26 mAb but not anti-IL-17A mAb completely inhibited the expression of *CXCL1*, *CXCL8*, and *IL1A* (Fig. 3 F), confirming the specific role of IL-26 as a driver of pathogenic autoinflammation circuits.

### Neutrophil-derived IL-26 activates kerytinocytes to produce CXCL1, CXCL8, and IL-1β

**Figure 4.**
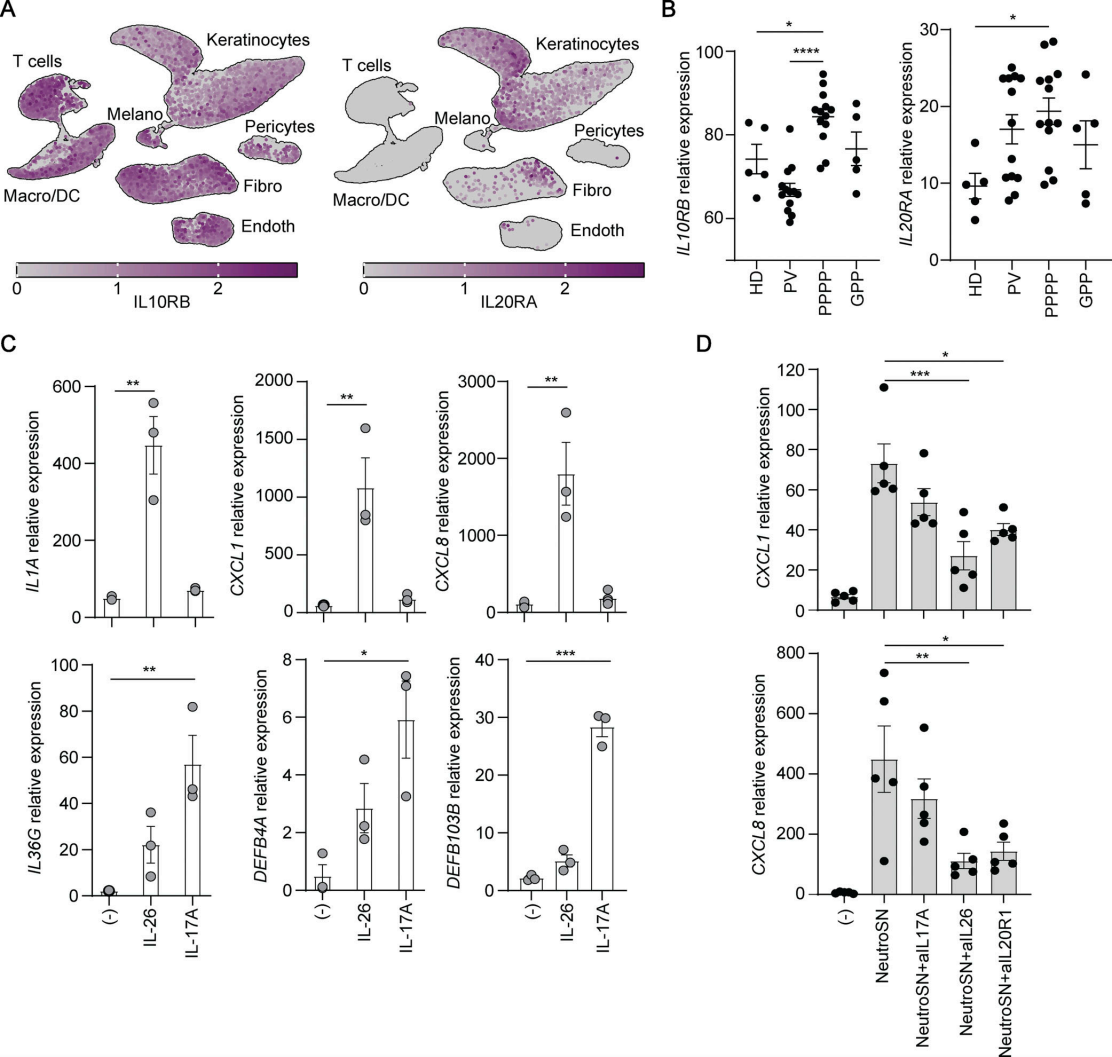
**Neutrophil-derived IL-26 drives autoinflammation by activating keratinocytes via the IL-26R. (A)** UMAP projection of the single cell transcriptomes of cells from lesional skin of three psoriasis patients colored according to the expression level of IL10RB (left) and IL20RA (right). **(B)** IL10RB (left) and IL20RA (right) mRNA expression in healthy donors (HD) (*n* = 5), PV (*n* = 13), PPPP (*n* = 13), and GPP (*n* = 5). Each data point represents a different patient. **(C)** *CXCL1*, *CXCL8*, *IL1A*, *IL36G*, *DEFB4A*, and *DEFB103B* mRNA expression in primary human keratinocytes (NHEK cells) stimulated overnight with IL-26 or IL-17A. Data are the mean ± SEM of triplicate wells and are representative of one out of three independent experiments. **(D)** *CXCL1* and *CXCL8* mRNA expression in primary human keratinocytes (NHEK cells) stimulated overnight with fMLP-activated neutrophil supernatants (NeutroSN, *n* = 5) in the presence of blocking antibodies against IL-17A, IL-26, or IL-20R1. Data are the mean ± SEM of five different neutrophil donors and representative of two independent experiments. B–D, P values (asterisks) were calculated by one-way ANOVA followed by Tukey’s test. *P < 0.05, **P < 0.01, ***P < 0.001, ****P < 0.0001.

IL-26 signals through the IL-26 heterodimer receptor composed of IL10R2 and IL20R1 and predominantly expressed by keratinocytes (Fig. 4 A) in both plaque-type and pustular psoriasis (Fig. 4 B). To investigate whether IL-26R–mediated keratinocyte activation could induce an autoinflammation signature, we stimulated primary keratinocytes with recombinant IL-26. As expected, IL-26 induced high expression levels of *CXCL1*, *CXCL8*, and *IL1A* expression but not expression of *DEFB4A*, *DEFB103B*, and *IL36G* (Fig. 4 C). By contrast, recombinant IL-17A did not induce this autoinflammation signature, but induced strong expression of *DEFB4A*, *DEFB103B*, and *IL36G* (Fig. 4 C). Stimulation of keratinocytes with supernatants of activated neutrophils also induced the autoinflammation signature with high expression of *CXCL1* and *CXCL8*, and this induction was blocked by inhibition of IL-26 but not IL-17A (Fig. 4 D), demonstrating that neutrophils induce autoinflammation circuits in keratinocytes via the release of IL-26. Activation of keratinocytes occurred via the IL26R, as the addition of neutralizing anti-IL-20R antibodies largely inhibited the induction of *CXCL1* and *CXCL8* in keratinocytes (Fig. 4 D).

### Neutrophil-derived IL-26 perpetuates neutrophil activation by forming microbial DNA complexes that trigger TLR9 activation

**Figure 5.**
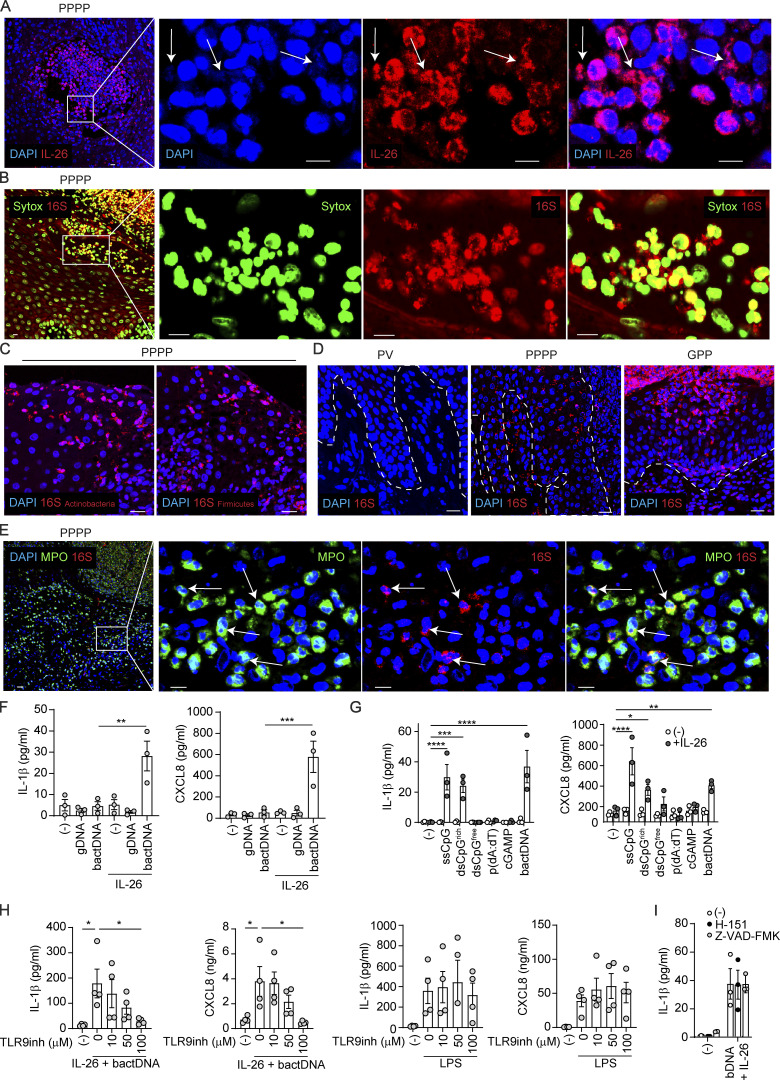
**Neutrophil-derived IL-26 perpetuates neutrophil activation by forming microbial DNA complexes that trigger TLR9 activation. (A)** Confocal image of a PPPP skin lesion stained for IL-26 (red) and DNA (blue) (left). Higher magnification images and individual stainings are shown (right). White arrows indicate extracellular DNA complexed with IL-26. Images are representative of five patients. Scale bars are 10 μm. **(B)** Confocal image of a PPPP skin lesion stained for 16S rDNA (red) and DNA (SYTOX, green) (left). Higher magnification images and individual staining are shown (right). Images are representative of five patients. Scale bars are 10 μm. **(C)** Confocal images of PPPP skin lesions stained for Actinobacteria (left) or Firmicutes (right) 16S rDNA (red) by FISH. Images are representative of 5 patients. Scale bars are 20 μm. **(D)** Confocal images of PV, PPPP, and GPP skin lesions stained for 16S rDNA (red) by FISH. Images are representative of five patients. Scale bars are 20 μm. **(E)** Confocal image of a PPPP skin lesion stained for MPO (green), 16S rDNA (red), and DNA (blue) (left). Higher magnification images and individual staining are shown (right). White arrows indicate MPO^+^ neutrophils containing 16 rDNA^+^ bacterial DNA. Images are representative of five patients. Scale bars are 20 μm (left image) and 10 μm (right images). **(F)** IL-1β and CXCL8 release by blood-isolated neutrophils stimulated overnight with genomic human DNA (gDNA) or bacterial DNA (bactDNA) complexed or not with IL-26. P values (asterisks) were obtained with one-way ANOVA followed by Tukey’s test. **P < 0.01, ***P < 0.001. **(G)** IL-1β and CXCL8 release by blood-isolated neutrophils stimulated overnight with single-stranded or double-stranded CpG-containing DNA targeting TLR9 (ssCpG, dsCpG^rich^), double-stranded CpG-free DNA (dsCpG^free^), poly(dA:dT) targeting AIM2 and cGAS-STING, cGAMP targeting STING, or bacterial DNA (bactDNA) complexed or not with IL-26. P values (asterisks) were obtained with one-way ANOVA followed by Tukey’s test. *P < 0.05, **P < 0.01, ***P < 0.001, ****P < 0.0001. **(H)** IL-1β and CXLC8 release by blood-isolated neutrophils stimulated overnight with bacterial DNA complexed with IL-26 (left) or LPS (right) in the presence of increasing concentrations of a TLR9 inhibitor. P values (asterisks) were obtained with one-way ANOVA followed by Tukey’s test. *P < 0.05. **(I)** IL-1β release by blood-isolated neutrophils stimulated overnight with bacterial DNA complexed with IL-26 in the presence or not of the STING inhibitor H-151 or the pan-caspase inhibitor Z-VAD-FMK.

**Figure S4.**
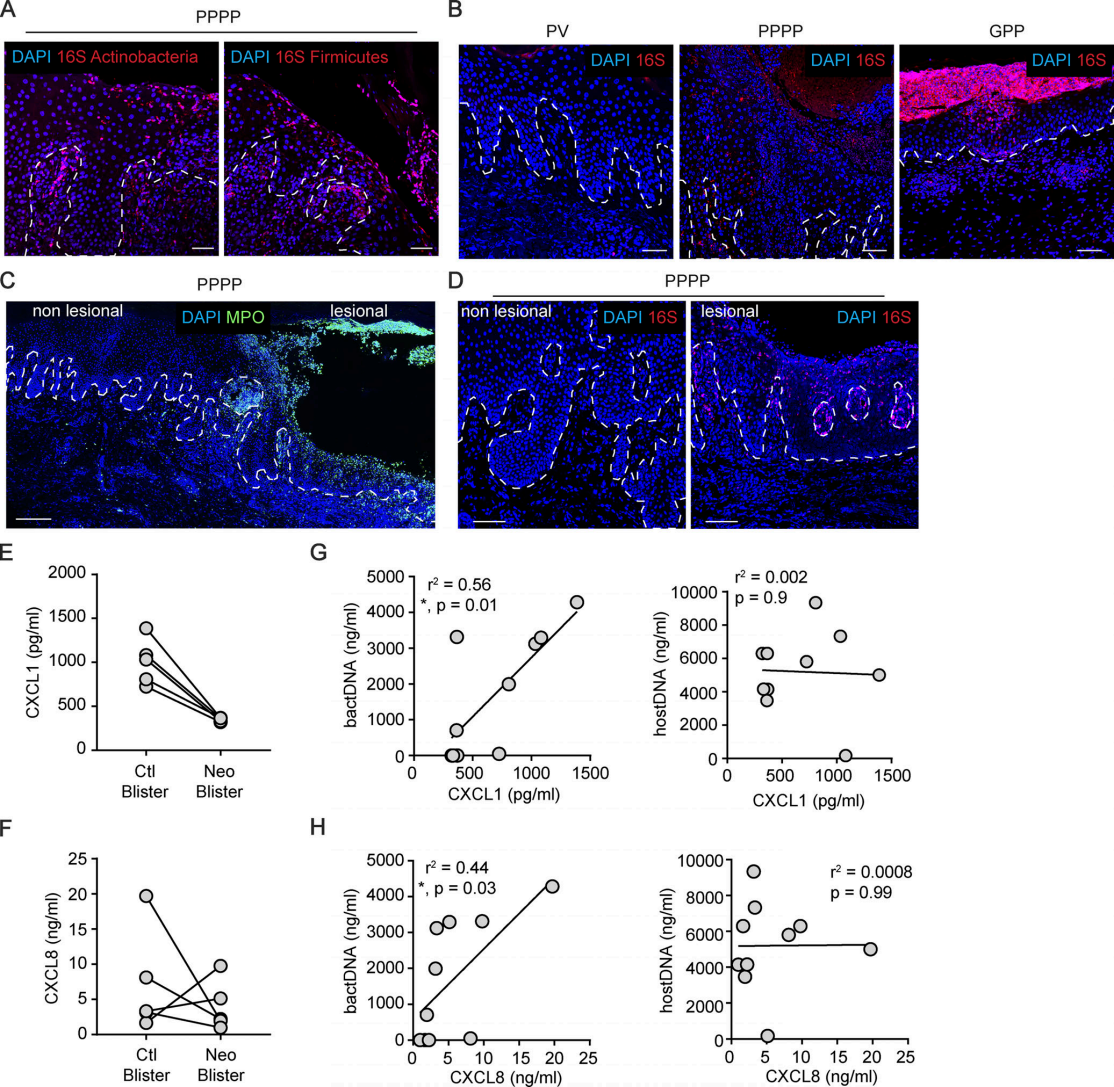
**Microbiota-derived DNA is present in skin lesions of pustular psoriasis as well as injured skin in association with CXCL1 and CXCL8 expression. (A)** Confocal images of PPPP skin lesions stained for Actinobacteria (left) or Firmicutes (right) 16S rDNA (red) by FISH. Images are representative of five patients. Scale bars are 50 μm. **(B)** Confocal images of PV, PPPP, and GPP skin lesions stained for 16S rDNA (red) by FISH. Images are representative of five patients. Scales bars are 50 μm. **(C)** Confocal image of PPPP skin stained for MPO (green) showing non-lesional and lesional areas. Images are representative of five patients. Scale bar is 200 μm. **(D)** Confocal images of non-lesional (left) and lesional (right) PPPP skin stained for 16S rDNA (red) by FISH. Images are representative of five patients. Scale bars are 100 μm. **(E and F)** Amount of CXCL1 (E) and CXCL8 (F) detected in skin blister fluids from vehicle-treated (Ctl) and Neosporin-treated (Neo) arms (*n* = 5). **(G and H)** Correlation between the levels of CXCL1 (G) and CXCL8 (H) and the amounts of bacterial DNA (left) and host-derived DNA (right). Pearson correlation coefficient and a two-tailed statistical significance are given (*n* = 10).

Because we have previously shown that IL-26 is also a potent trigger of innate immunity via binding extracellular DNA (Meller et al., 2015), we investigated whether extracellular DNA complexed with IL-26 was present in pustular psoriasis. We found abundant DAPI^+^ DNA fragments in the extracellular space that all costained for IL-26, indicating that they are IL-26–DNA complexes (Fig. 5 A). To further assess the origin of the extracellular DNA in the complexes, we used a SYTOX dye, which stains DNA fragments from host and bacteria, and the FISH technique with universal probes targeting 16S ribosomal DNA (rDNA) to visualize bacterial DNA. We found that all extracellular DNA complexes were 16S positive, indicating that they were of microbiota origin, similar to our observations in skin wounds (Fig. 5 B) (Di Domizio et al., 2020). Probes specific for Firmicutes and Actinobacteria 16S demonstrated that the microbial DNA was derived from multiple species (Fig. 5 C and Fig. S4 A). Interestingly, we were unable to detect live bacteria by gram-staining nor by culturing the tissue ex vivo (not shown), suggesting that the detected microbial DNA consisted of extracellular complexes generated in the context of bacterial killing by IL-26 (Meller et al., 2015). These bacterial DNA complexes were found in both lesional PPPP and GPP, associated with high numbers of neutrophils, but were not found in non-pustular psoriasis nor in non-lesional skin of pustular psoriasis (Fig. 5 D and Fig. S4, B–D). A closer look at these DNA complexes within cells showed that a large number was also present within MPO^+^ neutrophils but not inside other cells (Fig. 5 E), suggesting uptake of the extracellular bacterial DNA–IL-26 complexes by neutrophils. To investigate the consequences of this internalization, we stimulated purified neutrophils with IL-26–bacterial DNA complexes or IL-26 and bacterial DNA alone, as controls. We found that only IL-26–bacterial DNA complexes but not IL-26 and bacterial DNA alone induced strong activation of neutrophils with the specific release of IL-1β and CXCL8 (Fig. 5 F), but not other proinflammatory cytokines such as TNF or IL-17A (not shown). These findings are in line with the ability of IL-26 to transport complexed extracellular DNA into intracellular compartments with activation of intracellular DNA receptors (Meller et al., 2015). Surprisingly, genomic (mammalian) DNA fragments were unable to activate neutrophils even when complexed with IL-26 (Fig. 5 F). One major difference between bacterial and genomic (mammalian) DNA is the presence of high numbers of unmethylated CpG islets that activate TLR9. Because neutrophils express TLR9 (Hayashi et al., 2003), we tested the capacity of IL-26–DNA complexes containing specific unmethylated DNA sequences known to bind TLR9 (single-stranded and double-stranded CpG containing DNA) to activate neutrophils. By comparison, we assessed neutrophil activation by DNA sequences known to target cGAS-STING or AIM2 (all double-stranded DNA sequences, irrespective of CpG content) and by the STING-specific agonist cyclic guanosine monophosphate–adenosine monophosphate (cyclic GMP-AMP, cGAMP). We observed that neutrophil activation with release of IL-1β, CXCL8 (Fig. 5 G), and NET formation (not shown) was induced exclusively by DNA sequences containing CpG, both single-stranded and double-stranded, when complexed with IL-26, indicating that neutrophils are activated through TLR9. In fact, the selective TLR9 inhibitor TLR9-IN-1 blocked cytokine release induced by IL-26–DNA complexes but not the release induced by the TLR4 agonist LPS (Fig. 5 H). Furthermore, both the STING inhibitor H-151 and the pan-caspase inhibitor that blocks AIM-2 inflammasome activity did not inhibit the release of IL-1β, confirming that neutrophils detect these complexes through TLR9 and not through cytosolic DNA sensors (Fig. 5 I). We did not observe any type I IFN induction in neutrophils stimulated by these complexes in line with the lack of IFN signature in pustular psoriasis (not shown). To further confirm the requirement for microbiota-derived bacterial DNA in driving the autoinflammation process in vivo, we took advantage of the suction blister model described in Fig. 2 (Di Domizio et al., 2020). This model not only allows in vivo assessment of the proteins released by skin-infiltrating neutrophils (Di Domizio et al., 2020) but also allows dissection of the role of microbial components in neutrophil activation, as topical Neosporin treatment of the skin prior to blister induction can reliably deplete the skin microbiota (Di Domizio et al., 2020). We found blister fluids contained large amounts of neutrophil-derived CXCL1 and CXCL8 and observed their expression was largely abrogated when the microbiota was depleted (Fig. S4, E and F). Moreover, the levels of CXCL1 and CXCL8 significantly correlated with the amount of microbial 16S rDNA and not host-derived 18S rDNA in the blister fluid (Fig. S4, G and H), confirming that the autoinflammation process requires the presence of the skin microbiota and is driven by microbial DNA. Altogether our data suggest that IL-26–bacterial DNA complexes generated in the context of microbiota killing by IL-26 perpetuate neutrophil activation via TLR9, leading to the amplification of the autoinflammatory loop.

## Discussion

By comparing psoriasis subtypes, we identify a central role of IL-26 in driving and amplifying neutrophil-rich autoinflammation of the skin. We show that IL-26 is constitutively present in neutrophils and abundantly released from granular stores upon activation. Neutrophil-derived IL-26 triggers hallmark genes of autoinflammation including IL-1 cytokines and neutrophil recruiting chemokines, thereby contributing to pustule formation and disease activity in psoriasis.

**Figure S5.**
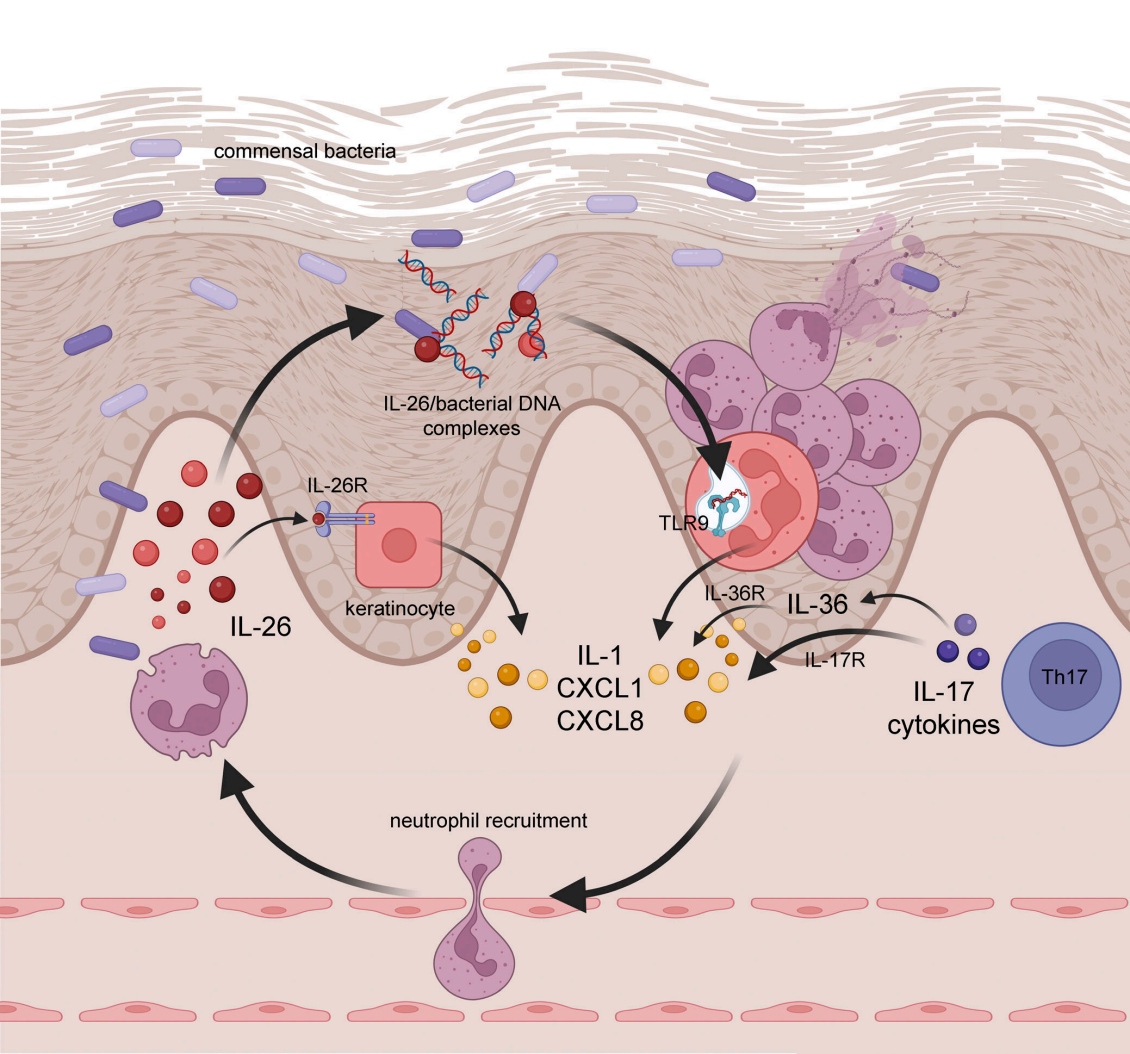
**Neutrophil-derived IL-26 triggers a self-sustaining pathogenic loop that drives autoinflammation in pustular psoriasis.** Skin-infiltrating neutrophils release IL-26, which activates keratinocytes to produce neutrophil-recruiting CXCL1 and CXCL8 chemokines along with inflammatory IL-1 cytokines. In pustular psoriasis, IL-26 also forms complexes with commensal bacterial DNA present in skin lesions, leading to a continuous activation of neutrophils and the release of neutrophil-recruiting chemokines. Th17 cells producing IL-17 cytokines induce some levels of CXCL1 and CXCL8 either directly, or via the induction of IL-36, and membrane expression of its receptor in keratinocytes. The IL-26–driven pathway provides a self-sustaining loop that amplifies neutrophil recruitment, autoinflammation, and leads to the development of pustular disease phenotype. Schematic was created with https://BioRender.com.

Our study describes two complementary IL-26–driven mechanisms underlying the ability of neutrophils to trigger autoinflammation. First, neutrophil-derived IL-26 activates keratinocytes via the IL-26R, leading to the epidermal expression of IL-1α, IL-1β, CXCL1, and CXCL8. Second, neutrophil-derived IL-26 activates newly recruited neutrophils by forming DNA complexes that are internalized to trigger TLR9 activation, leading to additional release of IL-26 and production of IL-1β and CXCL8 by neutrophils. Thus, IL-26 represents a key link between neutrophil infiltration and keratinocyte activation and triggers a self-sustaining amplification loop that continuously activates neutrophils (Fig. S5). It is possible that, beyond neutrophils, IL-26–DNA complexes also activate monocytes and macrophages to produce IL-1β via intracellular DNA sensors such as the inflammasome AIM2, as previously described (Poli et al., 2017).

Neutrophils are known to secrete cytokines such as IL-6 (Zimmermann et al., 2015), TNF (Dubravec et al., 1990), IL-1β (Cassatella, 1995), and IL-23 (Cassatella et al., 2019), but these cytokines are typically generated in mature neutrophils by de novo synthesis involving mRNA transcription (Beaulieu et al., 1995). Our study now finds that IL-26 is exclusively transcribed at early stages of granulopoiesis and subsequently stored as preformed protein in neutrophil granules. While this process is atypical for cytokines, it is consistent with the antimicrobial function of IL-26 (Meller et al., 2015). In fact, other neutrophil antimicrobial molecules such as MPO and LL-37 are also synthetized at the promyelocytic stage of neutrophil differentiation and stored in primary granules like IL-26. The granular storage allows, on one hand, the rapid release of antimicrobial effectors without the need for de novo synthesis, and, on the other hand, the regulated release of potentially cytotoxic molecules.

A particularly surprising observation is that the DNA in the IL-26–DNA complexes is of bacterial origin and not host derived, challenging the current view of autoinflammation as a complete sterile process. The generation of IL-26–bacterial DNA complexes is the result of bacterial killing by IL-26 and other neutrophil-derived antimicrobial proteins (Meller et al., 2015), most likely directed against multiple species in the skin microbiota and not against a single colonizing strains, as both Firmicutes and Actinobacteria 16S DNA sequences were detected in the complexes. Although NET formation with the presence of IL-26–selfDNA complexes also occurs, there is a preferential formation of IL-26–microbial DNA complexes likely dictated by the availability of the skin microbiota. This is in line with a previous report showing that pustules in PPPP contain abundant levels of commensal microbiota 16S rDNA (Masuda-Kuroki et al., 2018). Because the acrosyringium, the primary site of pustule formation in psoriasis (Murakami et al., 2010), is also the site where the skin microbiota penetrates the skin and descends into eccrine glands (Nakatsuji et al., 2013), we speculate that neutrophils in pustular psoriasis continuously encounter and kill the skin microbiota in and around the damaged sweat duct, leading to the generation of IL-26–microbial DNA complexes.

Our study also shows that the microbial DNA–dependent autoinflammation process occurs physiologically in the context of skin wounds. While in injured skin, neutrophil exposure to the skin microbiota may be transient, neutrophils in pustular psoriasis appear to be overactivated, triggering tissue-damaging inflammation that leads to continuous microbiota exposure. The reasons for this neutrophil overactivity in pustular psoriasis are currently unknown. Two genetic mutations associated with pustular psoriasis have been recently described: AP1S3 (Setta-Kaffetzi et al., 2014) and MPO (Vergnano et al., 2021). AP1S3 destabilizes the clathrin adaptor AP-1 complex formation involved in trafficking between the Golgi apparatus and late endosomes/lysosomes (Setta-Kaffetzi et al., 2014). AP1S3 mutations in pustular psoriasis reduce degradation activity in lysosomes and may favor intracellular accumulation of bacterial DNA complexes in neutrophils, leading to sustained TLR9 activation. On the other hand, MPO mutations inhibit clearance of neutrophils by macrophages (Vergnano et al., 2021; Haskamp et al., 2020; Onitsuka et al., 2023) and may therefore prolong the presence of activated neutrophils in pustular psoriasis.

Another genetic defect associated with pustular psoriasis is the IL36RN mutation, which decreases activity of the IL-36R antagonist and increases IL-36R signaling, leading to expression of IL-1β and CXCL8 (Marrakchi et al., 2011; Onoufriadis et al., 2011). Increased lesional IL-36γ levels have also been described (Johnston et al., 2017) and a blocking anti-IL36R antibody has been recently approved for the treatment of generalized pustular psoriasis (Bachelez et al., 2021; Elewski et al., 2023). However, in our study IL-36γ protein levels were not found to be elevated in pustular forms of psoriasis and were not directly associated with either the autoinflammatory signature, the number of neutrophils in the skin, or the clinical disease activity score. In fact, in contrast to IL-26 levels, IL-36γ levels were rather associated with the lesional Th17 signature, which is consistent with our finding that IL-17 cytokines trigger expression of IL-36γ in both total skin and keratinocyte cultures. Because IL-17 cytokines can induce some expression of IL-1 cytokines and neutrophil-recruiting chemokines either directly (Carrier et al., 2011) or via the induction of IL-36 (Carrier et al., 2011), and membrane expression of its receptor (Ni et al., 2022), it may be possible that IL17R and IL36R signaling represents initial drivers of neutrophil recruitment, a process which subsequently becomes autonomous via an autoinflammatory loop mediated by IL-26 (Fig. S5).

The treatment of pustular psoriasis, in particular PPPP, remains an unmet medical need. Anti-IL-17A secukinumab (Mrowietz et al., 2019) and anti-IL-23 guselkumab (Terui et al., 2018) have yielded disappointing results in the treatment of pustular psoriasis, achieving only Palmoplantar Psoriasis Area and Severity Index 75 in 26.6% and 11.5–20.4% of patients at week 16, respectively. Furthermore, anti-IL-12/-23 ustekinumab (Bissonnette et al., 2014), anti-IL-1R anakinra (Cro et al., 2021), and anti-IL-36R spesolimab (Mrowietz et al., 2021) showed no significant superiority to a placebo in a randomized controlled trial. Thus, blockade of IL-26 or inhibition of IL-26-mediated autoinflammation pathways may represent promising future strategies for the treatment of palmoplantar pustular psoriasis.

In conclusion, our work identifies constitutive IL-26 expression by neutrophils and demonstrates the role of high levels of IL-26 released by neutrophils in triggering the pathogenic autoinflammation gene program in pustular psoriasis. We show that this occurs via the ability of IL-26 to activate the IL26R in keratinocytes, and to kill the skin microbiota in pustular psoriasis, leading to the generation of immunogenic IL-26–DNA complexes. Our data provide a major advance in understanding the mechanisms that drive autoinflammation and point to IL-26 as a potential target for therapy of pustular psoriasis and potentially other autoinflammation-related diseases.

## Materials and methods

### Human samples

Studies were approved by the institutional review board of the Lausanne University Hospital and the local ethics committee, in accordance with the Helsinki Declaration (study 2019-01522). Lesional skin biopsies were performed in pustular psoriasis (PPPP, GPP, and ACH) and PV patients after informed consent was obtained. Clinical diagnosis was confirmed histologically. None of the patients were on systemic therapy and had discontinued topical corticosteroid treatment at least 2 wk prior to the biopsy. Comprehensive information on the year of disease onset, presence of concomitant plaque psoriasis, joint involvement, and location of biopsy are given in Table S1. In addition, for PPPP patients, disease activity was evaluated on photographs by five independent physicians using the physician global assessment (PGA) of erythema, pustules, and desquamation. The score is defined as PGA = 0 (none), PGA = 1 (slight), PGA = 2 (moderate), PGA = 3 (severe), and PGA = 4 (very severe) of the palm or sole that was biopsied. For human healthy skin, leftover material was obtained from excisional surgeries and 6-mm punch biopsies were performed. Control skin samples for transcriptomic analysis included pyoderma granulosum (PG), dissecting cellulitis (DC), and hidradenitis suppurativa (HS) and were obtained from the Dermatology Biobank at the Lausanne University Hospital. Blood tubes (9 ml EDTA) and buffy coats from healthy donors were obtained from the Interregional Blood Transfusion Center, Bern, Switzerland. For blister fluid analysis, residual material from the study 429/15, including six healthy volunteers, was used (Di Domizio et al., 2020). Briefly, skin blisters were induced by a vacuum pump on forearms, one pretreated topically with Neosporin cream to deplete the skin microbiota and the other treated with vehicle control cream.

### Human skin blister fluid analysis

Blister fluids derived from suction blisters of forearms treated topically with Neosporin cream or control cream were centrifuged for 5 min at 1,500 rpm. The supernatants were harvested for IL-26 measurement by ELISA (Cusabio Biotech). The cellular fraction was analyzed by flow cytometry (BD FACSCalibur) using the following antibodies: CD45-PerCP-Cy5.5 (BD), CD11b-APC or -FITC (eBiosciences), CD15-FITC (BD), Siglec8-PE (Biolegend), CD14-PE (BD), CD3-PE (BD), CD123-PE (BD), and BDCA4-APC (Miltenyi). Neutrophils were identified as CD45^+^CD11b^+^Siglec8^−^CD15^+^, eosinophils as CD45^+^CD11b^+^Siglec8^+^CD15^+^, monocytes as CD45^+^CD11b^+^CD14^+^CD15^−^, plasmacytoid dendritic cells (pDC) as CD45^+^CD11b^−^CD123^+^BDCA4^+^, and T cells as CD45^+^CD3^+^CD11b^−^.

### Neutrophil and Th17 cells isolation and stimulation

Human peripheral blood mononuclear cells (PBMCs) were obtained by centrifugation on Ficoll gradient (GE Healthcare). Neutrophils were isolated using the EasySep Direct Human Neutrophils Isolation Kit, and Th17 cells were isolated using the EasySep Human Th17 Cell Enrichment Kit II following the manufacturer’s instructions (StemCell). Cell purity was assessed by flow cytometry using CD16-PE (BD) and CD66b-APC (eBioscience) staining of neutrophils (purity >95%), and CD4-FITC, CXCR3-APC (eBioscience), and CCR6-PE (BD) staining of Th17 cells (purity >95%). For neutrophil stimulation, cells were plated at 10^6^ cells/ml and stimulated with human recombinant IL-6 (20 ng/ml; R&D Systems), IL-1β (50 ng/ml; R&D Systems), IL-23 (50 ng/ml; PreproTech), TNF (10 ng/ml; PreproTech), IFNα (10^3^ U/ml; PreproTech), Pam_2_CSK_4_ (1 μg/ml), LPS (1 μg/ml; LabForce), fMLP (1 μg/ml; Sigma-Aldrich), R848 (1 μg/ml; Invivogen), CpG-B (CpG-ODN 2006, 1 μM; Sigma-Aldrich), CpG-plasmid (pCpG-rich, 1 μM pBR322; Thermo Fisher Scientific), CpG-free-plasmid (pCpG-free, 1 μM pCpGfree-mcs; Invivogen), poly(dA:dT) (1 μg/ml; Invivogen), 2′3′-cGAMP (cGAMP, 4 μg/ml; Invivogen), genomic or bacterial DNA (1 μg/ml). In some instances, IL-26 (10 μg/ml, monomer; R&D systems) was complexed with the different oligonucleotides for 10 min before adding to the cells. In some experiments, neutrophils were pretreated with 10–100 μM TLR9 inhibitor (TLR9-IN-1; MedChemExpress) or 5 μM cytochalasin B (Sigma-Aldrich) for 1 h prior to cell stimulation. To confirm degranulation of neutrophils, cells were analyzed for the expression of CD63 by flow cytometry using CD63-APC antibody (Biolegend). For NETs induction, neutrophils were plated at 10^6^ cells/ml on poly-*L*-lysin (Sigma-Aldrich) coated coverslips, incubated for 1 h at 37°C for adherence, and then stimulated with LPS for 3 h at 37°C. For Th17 cell stimulation, cells were plated at 10^6^ cells/ml and stimulated with human recombinant IL-1β (50 ng/ml; R&D Systems) or with ImmunoCult Human CD3/CD28 T cell Activator (StemCell). For all experiments, supernatants were collected at different time points after stimulation to measure IL-26 (Cusabio) and LL-37 (AVIVA Systems) by ELISA, or IL-1β, CXCL8, and TNF by cytometric bead array (BD Biosciences).

### Neutrophil differentiation

CD34^+^ cells were isolated from healthy donors PBMC with the MACS CD34 Progenitor Cell Isolation Kit (Miltenyi) followed by FACS sorting of c-kit^+^ CD34^+^ cells. Progenitors were plated and grown for 3 days in IMDM with GlutaMAX I and 25 mM HEPES (Life Technologies) supplemented with 10% FBS and with 1% penicillin-streptomycin (Sigma-Aldrich), SCF (100 ng/ml; PreproTech), and IL-3 (100 ng/ml; PreproTech) at a concentration of 10^4^ cells/ml. After 3 days, medium was supplemented with G-CSF (100 ng/ml; StemCell). Every 48 h, cells were collected for analysis, medium was renewed, and cells were maintained at 10^5^ cells/ml.

### Western blot analysis

Neutrophils were lyzed with Pierce RIPA Buffer (Thermo Fisher Scientific) following the manufacturer’s instruction. Protease Inhibitor Cocktail (Sigma-Aldrich) was added to prevent protein degradation. 20 μg of the lysate was loaded on a 4–20% Tris-Glycine gel (Invitrogen) and ran in Novex Mini Cell (Invitrogen) following the manufacturer’s instructions. Human recombinant IL-26 (10 ng) was also loaded for control. Proteins were transferred on Amersham Hybond polyvinylidene difluoride membrane (GE Healthcare) and blocked for 1 h at room temperature with Tris-buffered saline with 0.1% Tween 20, 5% milk. The membrane was probed with primary antibodies (mouse anti-IL-26, clone 84, 1:1,000; or mouse anti-actin, BD, 1:5,000) and incubated overnight at 4°C. After washing, the membrane was probed with the corresponding secondary antibody HRP conjugated (rabbit anti-mouse HRP, 1:10,000; Dako) and incubated 1 h at room temperature. After washing, the membrane was revealed with WesternBright Sirius (Advasta) using Fusion Fx (Vilber Lourmat).

### TEM

Fresh blood-isolated neutrophils were fixed with 2% formaldehyde and 0.2% glutaraldehyde (EMS) in phosphate buffer (PB) 0.1M (Sigma-Aldrich) overnight. Cells were then spun down in prewarmed gelatin 12% (Sigma-Aldrich) in PB 0.1M and pellets were then gelled on ice and cryoprotected by immersion in 2.1 M sucrose overnight. The pellets were then finally cryoimmobilized in liquid nitrogen. Ultrathin cryosections of 100 nm were cut on a Leica UC6-FC6 microtome (Leica Mikrosysteme GmbH) and picked up using sucrose 2.3 M droplet and deposited on Formvar carbon-coated nickel grids (EMS). Immunogold labeling was performed using mouse anti-IL26, clone 84 (4 μg/ml), rabbit anti-MPO (4 μg/ml; Dako), rabbit anti-Lactoferrin (4 μg/ml; Abcam), and rabit anti-MMP9 (4 μg/ml; Abcam) together with goat anti-rabbit IgG coupled to 10-nm colloidal gold particles (EMS) and goat anti-mouse IgG coupled to 15-nm colloidal gold particles (EMS). Samples were then stained for contrasting with a mixture of 1.8% of methylcellulose (Sigma-Aldrich) and 0.4% of uranyl acetate (Sigma-Aldrich) in H_2_O. Micrographs were taken with a transmission electron microscope Philips CM100 (Thermo Fisher Scientific) at an acceleration voltage of 80 kV with a TVIPS TemCam-F416 digital camera (TVIPS GmbH). Large montage alignment was performed using the Blendmont command-line program from the IMOD software (Kremer et al., 1996).

### Immunofluorescence analysis

For cells, blood-isolated neutrophils or skin-infiltrating cells or neutrophil differentiating cells were plated on poly-*L*-lysin (Sigma-Aldrich) coated slides for 1 h at 37°C. Cells were fixed with 4% PFA for 10 min at room temperature and stained with goat anti-human IL-26 (R&D Systems) and mouse anti-human LL37 (Santa Cruz Biotechnology) at a concentration of 1 μg/ml for 2 h at room temperature in Permeabilization Buffer (Life Technologies). Cells were then stained with chicken anti-goat AF488 conjugated antibody (1:500; Invitrogen) and a goat anti-mouse AF546 or a donkey-anti-mouse AF546 (1:500; Invitrogen) for 1 h at room temperature. Neutrophils stained with one or the other primary antibody and isotypes, followed by both secondary antibodies, were used as control. Slides were then washed and mounted with Fluorescence Mounting Medium with DAPI (Abcam) and sealed. Images were acquired with a Zeiss LSM 700 confocal microscope and analyzed with Zen 2010 software. For skin sections, formalin-fixed paraffin-embedded (FFPE) blocks were cut into 6-μm sections and placed on slides. Sections were first deparaffinized and rehydrated, and then heat-induced epitope retrieval was performed and sections were permeabilized with PBS 0.01% Triton. Samples were stained with goat anti-human IL-26 (R&D Systems), rabbit anti-human MPO (Dako), or mouse anti-human CD3 (Dako) for 2 h at room temperature. Sections were then stained with chicken anti-goat AF488 conjugated antibody (Invitrogen) and with donkey anti-rabbit AF546 (invitrogen) or chicken anti-mouse AF546 (Invitrogen) for 30 min at room temperature. Images were acquired with a Zeiss LSM 700 confocal microscope and analyzed with Zen 2010 software. For cell quantification, slides were digitalized using the PANNORAMIC 250 Flash digital scanner (3DHISTECH Ltd.), and cell types were quantified using the QuantCenter plugin 2.2 of Caseviewer 2.4 software.

### FISH staining

FFPE skin sections were used to perform FISH analysis using the Vysis IntelliFISH Universal FFPE Tissue Pretreatment and Wash Reagents Kit (Abbot) according to the manufacturer’s instructions. Basically, skin sections were first deparaffinized and rehydrated, then antigen retrieval was performed by incubating slides in Protease IV solution for 20 min at 37°C. Sections were then dehydrated and air-dried followed by denaturation for 10 min at 75°C and hybridization of 4 ng/μl probes for 16 h at 37°C in Vysis IntelliFISH Hybridization Buffer (Abbot). Oligonucleotide probes (EUB338: 5′-GCT​GCC​TCC​CGT​AGG​AGT-3′) targeting 16S rDNA of most bacteria labeled with Alexa546 (Thermo Fisher Scientific) were used, and labeled probes designed with the reverse sequence of EUB338 were used as controls. Slides were then washed in post-hybridization wash buffer (Abbot) and mounted with ProLong Gold Antifade Mountant with DAPI (Thermo Fisher Scientific) and analyzed on a Zeiss LSM 700 confocal microscope.

### RNA extraction and real-time PCR analysis

For tissues, excised tissue was immediately snap-frozen in liquid nitrogen and stored at −80°C until RNA was isolated. For cells, dry pellets of cells were frozen and stored at −80°C until RNA was isolated. RNA was isolated using the TRIzol/chloroform method and a tissue homogenizer (Thermo Fisher Scientific). All isolated RNA had an A260/A280 value of ≥1.7 and RNA integrity was analyzed on a Fragment analyzer (Agilent). For reverse transcription-quantitative polymerase chain reaction (RT-qPCR), 2 μg RNA was used to generate cDNA using a commercial kit (QscriptT XLT cDNA Supermix; VWR). cDNA was used for each individual gene expression analysis using Taqman-based amplification on a QuantStudio 12K Flex Real-Time PCR System (Thermo Fisher Scientific) using the default Taqman standard protocol. Human Taqman probes used were *GAPDH*, *CXCL1*, *CXCL8*, *IL1A*, *IL1B*, *IL36G*, *DEFB4A*, and *DEFB103B*. Cycle threshold (Ct) values of *GAPDH* were used to calculate the relative expression of each gene using the Delta Delta CT method.

### NanoString analysis

The mRNA expression of 600 targets was analyzed with the nCounter Human Immunology V2 panel including 20 customized probes (Nanostring Technologies) on the nCounter platform (Nanostring Technologies) using 100 ng of RNA per skin sample. This commercial panel was extensively validated in-house for accuracy, repeatability, and reproducibility before analyzing the study samples. A quality check was run for each sample before including it in the analysis. Data were normalized and analyzed using ROSALIND (ROSALIND, Inc.). Basically, housekeeping probes to be used for normalization are selected based on the geNorm algorithm as implemented in the NormqPCR R library. Clustering of genes for the final heatmap of differentially expressed genes was done using the Partitioning Around Medoids method using the fpc R library that takes into consideration the direction and type of all signals on a pathway, the position, role, and type of every gene. The z-values of each gene were then calculated for the selected patients to generate heatmaps and determine specific classifiers. The autoinflammation gene signature score was calculated as the sum of z-values of *IL1A*, *IL1B*, *CXCL1*, and *CXCL8* for each patient.

### Data visualization of psoriatic skin single-cell RNA sequencing (scRNAseq)

scRNAseq data generated by Reynolds et al. (2021) were reanalyzed using processed datasets from nonlesional and lesional skin of three psoriasis patients (ArrayExpress database under accession number E-MTAB-8142). The gene counts matrix was analyzed with the R package Seurat v4.0 (Satija et al., 2015). The count matrix was normalized and scaled using the Seurat functions NormalizeData and ScaleData with all genes. A nonlinear dimensional reduction of the dataset was run to allow Uniform Manifold Approximation and Projection (UMAP) exploration of the data using the Seurat functions FindVariableFeatures (selection.method = “vst,” nfeatures = 7,500) and RunPCA followed by FindNeighbors (dims = 1:15), FindClusters (resolution = 0.5), and RunUMAP (dims = 1:15). The do_FeaturePlot function from SCpubr (Blanco-Carmona, 2022, *Preprint*) was used to generate UMAP plots colored according to the expression of IL20RA and IL10RB.

### Stimulation of skin explants

Healthy skin biopsies were first rinsed in PBS containing antibiotics (1% penicillin-streptomycin) and then cultured in 48-well plates in the presence of 200 μl of DMEM 10% FBS. Recombinant human IL-26 or IL-17A was then added to the biopsies at different concentrations (0, 0.1, 1, and 10 μg/ml) for 6 h. In some experiments, 10^6^ blood-isolated neutrophils, preactivated or not with 10^9^ heat-killed *Staphylococcus epidermidis* in 25 μl, were injected into the biopsy with a 30G-needle syringe. For PPPP patients’ skin, pustules containing 6-mm punch biopsies were cut into four pieces and one piece was snap-frozen to measure the baseline genes’ expression. The three remaining pieces were cultured as above overnight in the presence or not of 100 μg/ml blocking anti-IL-26 antibodies (Clone 84) or anti-IL-17A antibodies (secukinumab). For both healthy and patients’ skin, biopsies were then homogenized in Trizol to perform RNA extraction followed by RT-qPCR analysis as described above.

### Statistical analysis

Statistical analyses are described in each figure legend. For experiments combining several groups, an ordinary one-way ANOVA test was used. Significant differences between groups were determined by post-hoc Tukey’s multiple comparisons tests, unless specified otherwise, using GraphPad Prism 9.0 software. P > 0.05 was considered nonsignificant.

### Online supplemental material

Table S1 contains the clinical characteristics of patients used in this study. Fig. S1 shows an extended analysis of triggers inducing IL-26 release by neutrophils in vitro and the formation of NETs in vivo. Fig. S2 shows an extended analysis of IL-26 expression during in vitro granulopoiesis. Fig. S3 shows that the IL-17A–derived signature is not linked to the autoinflammation process in pustular psoriasis. Fig. S4 shows an extended analysis of the presence of microbial DNA in non-lesional and lesional skin of pustular psoriasis as well as healthy injured skin. Fig. S5 is a schematic illustrating the study’s main findings.

## Supplementary Material

Table S1 shows clinical characteristics of patients.

SourceData F2 is the source file for Fig. 2.

## Data Availability

The data underlying Figs. 1, 2, and 5 are available in the published article and its online supplemental material. The data underlying Fig. 3 are openly available in GEO DataSets at GSE255248. The data underlying Fig. 4 were derived from Reynolds et al. (2021) in ArrayExpress at https://www.ebi.ac.uk/arrayexpress/experiments/E-MTAB-8142.
