# Supplementary figures and images for: Human neutrophils drive skin autoinflammation by releasing interleukin (IL)-26
Source: J Exp Med. 2024 Mar 6;221(5):e20231464. doi: 10.1084/jem.20231464 (PMC10917069; doi:10.1084/jem.20231464)

Bright light

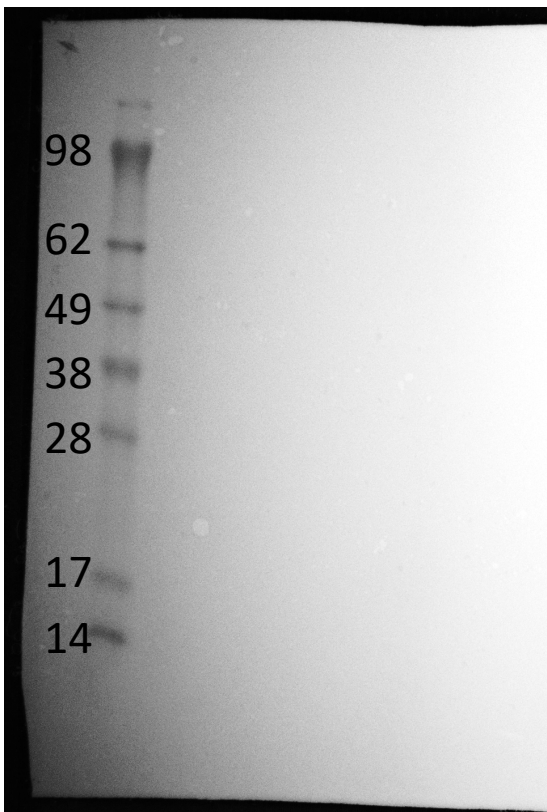

IB: IL-26

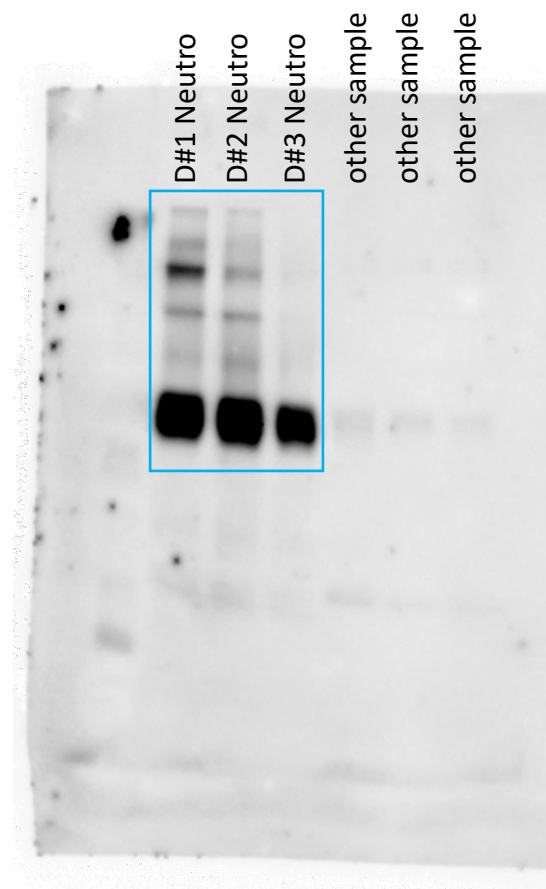

IB: actin

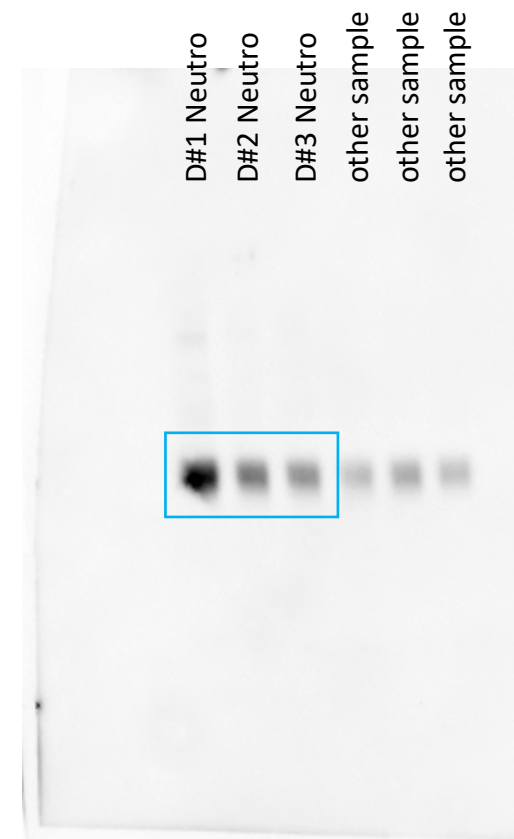

Supplement: SourceData F2 — is the source file for Fig. 2. [file JEM_20231464_SourceDataF2.pdf]
